# Carbapenem-resistant *Klebsiella pneumoniae* in Barbados: Driving change in practice at the national level

**DOI:** 10.1371/journal.pone.0176779

**Published:** 2017-05-25

**Authors:** Corey Forde, Bryan Stierman, Pilar Ramon-Pardo, Thais dos Santos, Nalini Singh

**Affiliations:** 1 Queen Elizabeth Hospital, Bridgetown, Barbados; 2 Children’s National Health System, Washington DC, United States of America; 3 Pan American Health Organization, Washington DC, United States of America; 4 George Washington University, Washington DC, United States of America; Ross University School of Veterinary Medicine, SAINT KITTS AND NEVIS

## Abstract

**Introduction:**

Carbapenem-resistant *Klebsiella pneumoniae* (CRKP) is of growing concern globally. The risk for transmission of antimicrobial resistant organisms across several continents to the Caribbean is a real one given its tourism industry. After a cluster of cases of CRKP were detected, several studies detailed in this report were initiated to better characterize the problem.

**Methods:**

A hospital-wide point prevalence study and active surveillance were performed at Queen Elizabeth Hospital (QEH) in Barbados in 2013 to assess the prevalence of CRKP infection/colonization. Following this, a 1-year longitudinal study measured the prevalence of CRKP isolates in the hospital and across all healthcare facilities in the country.

**Results:**

In 2013, eleven viable isolates of CRKP from cluster of cases were sent for molecular epidemiology studies. When sequenced, they were found to be the ST-258 clone. Identification of a cluster of cases of CRKP ST-258/512 clones indicated person-to-person transmission. In September 2013, the hospital-wide point prevalence study revealed 18% of patients (53/299) at the hospital were either colonized or infected with CRKP. The infection to colonization ratio was 1:7. Patients who were infected/colonized vs. non-colonized were older (64.7 vs. 48.7 years, p<0.0001), were hospitalized longer (42.5 days vs. 27 days, p = 0.0042), were more likely to have an invasive device (66% vs. 32%, p<0.0001), especially urinary catheters (55% vs. 24%, p<0.0001), and were more likely to have used antimicrobials within the prior 14 days (91% vs. 46%, p<0.0001). Specific antimicrobials, including fluoroquinolones and piperacillin-tazobactam, were significantly associated with infection/colonization. In 2014, the 12-month period prevalence of CRKP in Barbados was 49.6 per 100,000 population and of blood stream infections was 3.2 per 100,000 population.

**Conclusions:**

This point prevalence study identified patients at-risk of acquisition of CRKP and allowed QEH to implement interventions aimed at decreasing the prevalence of CRKP. Organization of a National and regional Infection Prevention and Control Committee in 2014 aimed to strengthen antimicrobial resistance surveillance programs across the English-speaking Caribbean were established.

## Introduction

**Fig 1:**
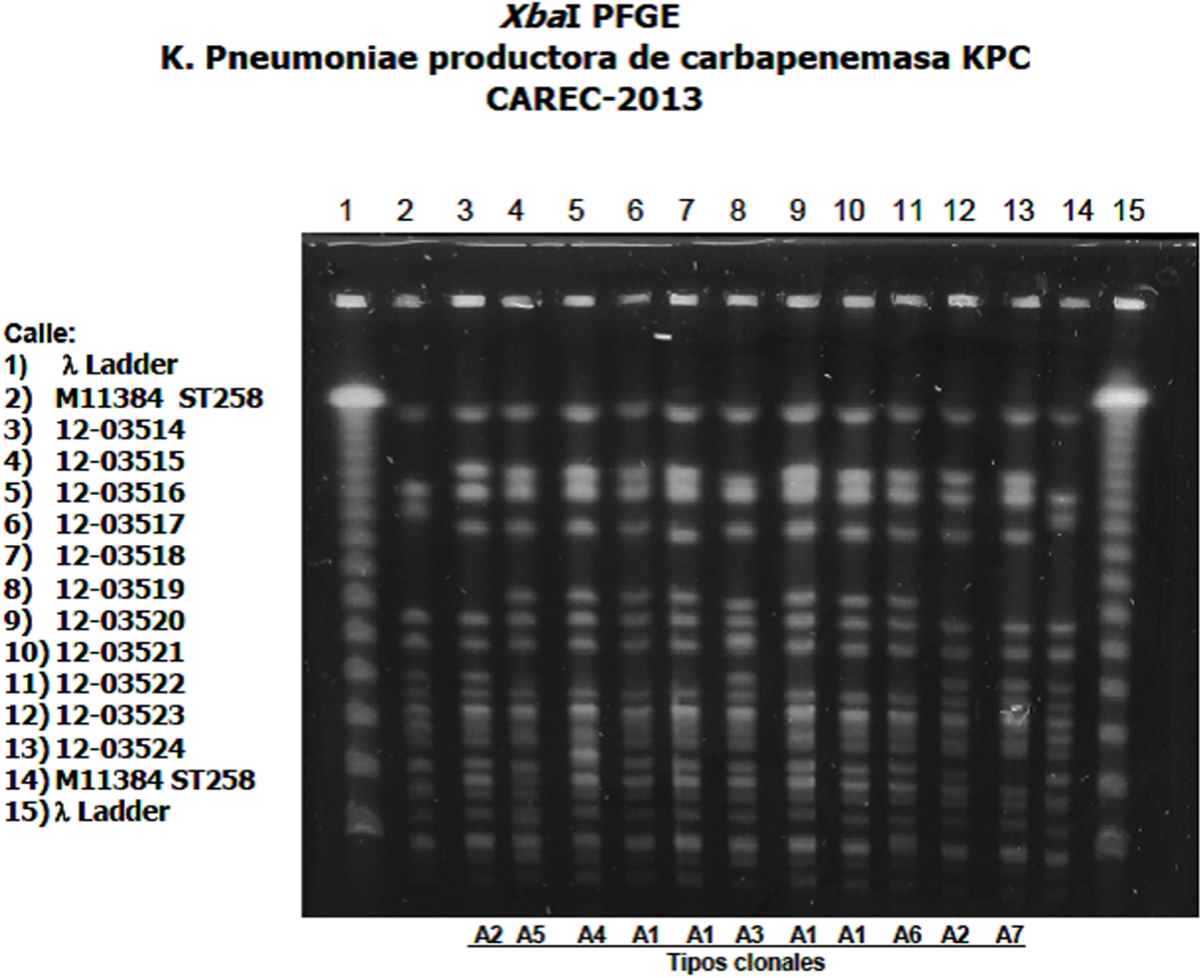
PFGE for molecular typing of selected samples showing similarities in band patterns across each sample.

Carbapenem-resistant *Klebsiella pneumoniae* (CRKP) is of growing concern globally with sporadic cases as well as epidemics reported in numerous countries spanning multiple continents across the world [1–7]. CRKP is a critical priority organism in the World Health Organization’s global priority list of antimicrobial resistant bacteria, which is meant to guide research, discovery and development of new antimicrobials, and global antimicrobial resistance surveillance [8,9]. CRKP is associated with increased morbidity and mortality in the hospital setting in part due to the limited treatment options available [3]. Varying risk factors have been associated with CRKP colonization, including organ or stem cell transplant, mechanical ventilation, tracheostomy, nasogastric tube, fecal incontinence, length of hospital stay, and exposure to certain antimicrobials [2,4,5,10,11]. A cluster of cases of CRKP was reported across multiple patient care units of the Queen Elizabeth Hospital (QEH) in Barbados, revealing concern for a widespread problem. The Ministry of Health in Barbados sought consultation from the Pan American Health Organization for support in further investigation. In 2013, molecular epidemiology of 11 of the 17 viable samples showed a dominant clone and the possibility of person-to-person transmission within a healthcare setting (Fig 1). The aim of this study is to report the point-prevalence of colonization/infection with CRKP at QEH and the subsequent results of a 1-year longitudinal study on the incidence of CRKP infections in the country.

## Materials and methods

### Setting

Queen Elizabeth Hospital is the only public hospital in Barbados and serves as the referral hospital for a population of 250,010 (119,926 males and 130,084 females). It is a 600-bed public hospital that averages an occupancy rate of 70%, with inpatient wards serving general acute care, intensive care (ICU), high dependency care, surgical care, obstetrics and gynecology, and pediatrics as well as outpatient services. The region's geriatric and psychiatric hospitals also send patients to QEH. It offers free medical services to all Barbadian nationals and caters medical services to several other countries within the English speaking Caribbean region, on demand. QEH Human Subject Research and Ethics Board approved the point prevalence study done in Sept 2013. Verbal consent was obtained at the time of taking the rectal swab for the point prevalence study. If patients refused or had some other reasoning for a lack of participation this was documented in the information sheet.

### Case definition

CRKP was defined using the Clinical Laboratory Standards Institute (CLSI) as isolation of *Klebsiella pneumoniae* 1) not susceptible to at least one of the following carbapenems: ertapenem, imipenem, meropenem, or doripenem and 2) resistant to all tested third-generation cephalosporins including ceftriaxone, cefotaxime, and ceftazidime [12]. A case was considered colonized when CRKP was isolated from the rectal swab and considered infected when the patient exhibited clinical signs of infection and culture revealed CRKP.

### Study design

After an initial investigation of a cluster of cases in 2013, a hospital-wide point prevalence study was conducted on a single day in September 2013 across 26 patient care units/wards, containing a total of 311 patients. A total of 299 patients were included in the analysis. On this day, there were 6 known cases of infection with CRKP in one unit, an isolation ward, as well as 1 case in the surgical intensive care unit. These known cases were included in the analysis. Eight people who refused rectal swab and 4 patients who were transferred off the unit at the time of swab collection were excluded from analysis. Upon completion of the point-prevalence study, surveillance was carried out for the 12-month period in 2014 to assess the prevalence of CRKP using the CDC’s case-based surveillance definition [13].

### Specimen and data collection

Medical staff were educated by the hospital infection prevention control (IPC) team on the correct procedure for rectal swab collection. Rectal swabs were then collected from each patient included in the study. Twenty-one link staff nurses and three IPC nurses on each ward were charged with obtaining verbal consent. Upon collection, swabs were transferred to the onsite microbiology laboratory for processing. CRKP was identified in the microbiology laboratory in Barbados by culture, using the Modified Hodge test. CLSI minimum inhibitory concentration breakpoints were used [12]. Patient data was collected on a standardized form with the following information obtained at the time of the rectal swab collection: age, gender, ward, ICU status, length of stay in hospital prior to date of swab collection, use of antimicrobials within 14 days prior to swab collection, use of invasive devices within 30 days prior to swab collection, and medical co-morbidities. Patients were classified as infected or colonized using the CDC surveillance definition [14]. Following the point-prevalence study, specimens positive for CRKP were tracked throughout QEH and other health care facilities in Barbados during the 12 month period in 2014. This data was used to calculate the prevalence of CRKP at QEH as well as in the population of Barbados. In 2013, 11 viable samples from the cluster of 17 cases from different units and patients were sent via the Caribbean Public Health Agency to the regional laboratory at Malbran National Institute of Infectious Diseases in Argentina where pulsed field gel electrophoresis (PFGE) was used for molecular typing (CHEF-DRIII). Tenover criteria were used to classify organisms as indistinguishable, closely related, possibly related or different [15]. Briefly, the restriction enzyme used was *Xba*I. The allelic variant of the KPC (*Klebsiella pneumoniae* carbapenemase) gene was determined by sequencing of the PCR product by the method of Sanger, using the BigDye Terminator Applied Biosystem methodology. The nucleotide sequence was analyzed using the DNASTAR computational software package, in addition to Chromas, ClustalX, and others programs with free access such as BLAST and PROSITE (National Center for Biotechnology Information, Bethesda, MD). The genetic environment of the KPC gene was determined by PCR mapping [16,17].

### Statistics

Data analyses were carried out with EpiInfo7.1.5 (Centers for Disease Control and Prevention, Atlanta, GA). The χ^2^ test or Fisher Exact test were used to evaluate associations between CRKP colonized patients and categorical variables. The Wilcoxon rank-sum test was used to evaluate associations between CRKP colonized patients and continuous variables. Binomial logistic regression was performed with all predictors to control for any confounding by other variables. EpiInfo uses the Newton-Raphson method for maximizing likelihood in logistic regression. Conditional mean imputation was used to average cases and non-cases separately and replace missing values for the logistic regression analysis. Incidence and prevalence measures were calculated using standard definitions.

## Results

During the hospital-wide point-prevalence study in September 2013 in 26 wards, a total of 53 (18%) patients were found to be colonized or infected and 246 (82%) were not colonized. Of these, 7 were active infections and 46 were colonized patients, giving an infection to colonization ratio of 1:7. Of the infected patients, 3 had bacteremia and 4 had urinary tract infections. One patient with a urinary tract infection progressed to have bacteremia. Several factors were significantly associated with colonization/infection with CRKP (Table 1). Patients who were colonized/infected were older, 64.7 years old, compared to those not colonized/not infected, 48.7 years old (p<0.0001). Patients who were colonized/infected on average were in the hospital longer, 42.5 days, vs. non-infected, 27 days (p = 0.0042). Invasive devices were also significantly associated with colonization/infection. Those colonized/infected were more likely to have an invasive device (p<0.0001), especially urinary catheters (p<0.0001), compared to those who were not colonized/not infected. Antimicrobials were used more often within the last 2 weeks (90.6% vs. 45.9%, p<0.0001) and in higher quantities (2.1 vs. 0.9, p<0.0001) in colonized/infected patients compared to controls.

**Table 1:** Characteristics of CRKP positive patients from point prevalence study.

Characteristics		Case (n = 53), n (%)	Non-Cases (n = 246), n (%)	p
**Gender**	**Female**	30 (56.6%)	153 (62.2%)	0.45
**Age (years)**	**Mean (Min, Median, Max)**	64.7 (26,65,95)	48.7 (0,49,102)	<0.0001^a^
**Length of Stay**	**Mean (Min, Median, Max)**	42.5 (1,15,746)	27.0 (1,8,410)	0.0042^a^
	**>10 days**	36 (67.8%)	113 (46.1%)	0.0040^a^
**Invasive Devices**	**Mean Number of Devices (Min, Median, Max)**	1.02 (0,1,5)	0.51 (0,0,5)	<0.0001^a^
	**Any Device**	35 (66.0%)	78 (31.7%)	<0.0001^a^
	**Urinary cath**	29 (54.7%)	60 (24.4%)	<0.0001^a^
	**Mechanical Ventilation**	2 (3.8%)	12 (4.9%)	1.00
	**Nasogastric Tube**	11 (20.8%)	29 (11.8%)	0.082
	**Invasive Vascular Line**	7 (13.2%)	18 (7.3%)	0.17
**Antimicrobials**	**On Antimicrobials**	48 (90.6%)	113 (45.9%)	<0.0001^a^
	**Mean Number of Antimicrobials (Min, Median, Max)**	2.1 (0,2,6)	0.9 (0,0,6)	<0.0001^a^
**Location**	**In Intensive Care Unit**	5 (9.4%)	1 (0.4%)	0.00077^a^

Min, Minimum; Max, Maximum; Urinary Catheter; Nasogastric Tube, Intensive Care Unit

^a^p ≤ 0.05 considered significant

The classes of antimicrobials used in colonized/infected vs. non-colonized/non-infected patients were variable with significant increases in usage of fluoroquinolones and piperacillin-tazobactam among those colonized/infected (p<0.05). Patients located in the High Dependency Unit were more likely to be colonized/infected than others (p = 0.0008). Comorbidities, diabetes, hypertension, chronic kidney disease, devices like, mechanical ventilation, NG tube, invasive vascular lines, and surgical drains were not found to be significantly associated with colonization/infection. After controlling for each variable through logistic regression analysis, older age (OR = 1.03, p = 0.016), length of stay >10 days (OR = 2.30, p = 0.034), presence of invasive devices (OR = 3.36, p = 0.0022), and use of antimicrobials (OR = 12.08, p<0.0001) remained significant predictors of colonization/infection with CRKP (Table 2). The crude mortality rate after 7 days from specimen collection was 130 per 1000 patients for cases that were colonized/infected compared to 70 per 1000 patients for those not infected.

**Table 2:** Logistic regression analysis of CRKP in point prevalence study.

Variable	Odds Ratio	95% Confidence Interval	P-Value
**Gender (Male = 0)**	0.86	(0.41–1.81)	0.69
**Age (Continuous variable)**	1.03	(1.00–1.05)	0.016^a^
**Length of Stay >10 Days (No = 0)**	2.30	(1.06–4.97)	0.034^a^
**Invasive Device Present? (No = 0)**	3.36	(1.55–7.28)	0.0022^a^
**Any Antimicrobial Used (No = 0)**	12.08	(4.35–33.50)	<0.0001^a^

^a^p ≤ 0.05 considered significant

Molecular typing was performed on 11 viable samples which were confirmed as carbapenemase producers by Modified Hodge test. Isolates showed similar Isolates showed similar susceptibility patterns. PFGE was used for molecular typing, utilizing the restriction enzyme *Xba*I, and revealed that samples were genetically related with 7 subtypes of the same clonal type based on analysis using the Tenover criteria [15]. The allelic variant of the KPC gene was established as KPC-3 in the transposon Tn4401a, which has been globally associated with the KPC-*K*. *pneumoniae* ST258 epidemic clone (Fig 1). All samples tested positive by PCR for PILV-like protein (pilv-l) and 10 of 11 were PCR prp positive. Sequencing allowed for molecular typing which revealed isolates were of the ST-258 clone.

**Fig 2:**
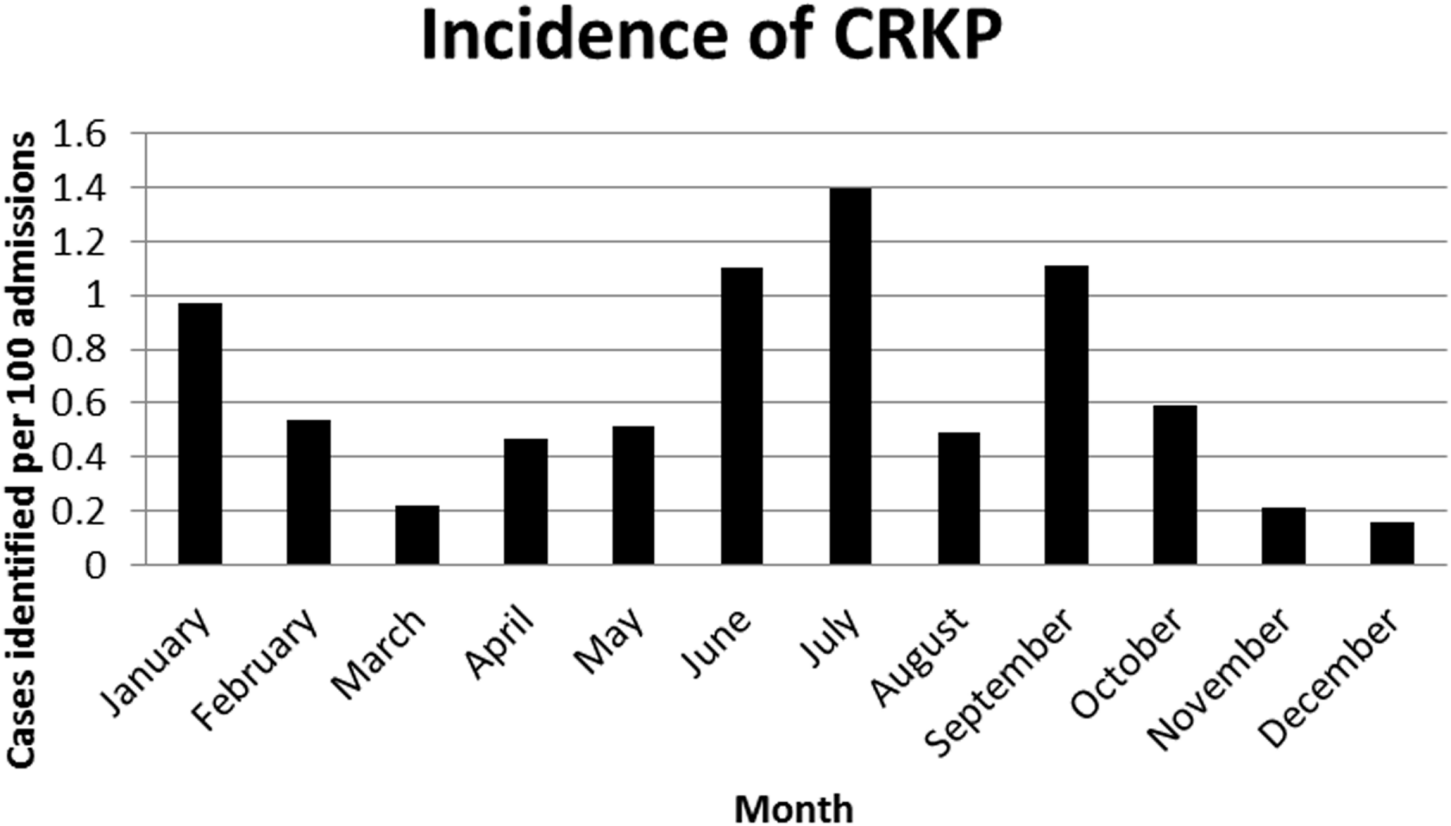
Incidence of CRKP in 2014.

The point prevalence study completed in September 2013 was followed by a 1-year longitudinal study of CRKP in 2014 at QEH and other Barbados health care sites. The peak incidence of CRKP occurred in the month of July with 1.4 infections per 100 admissions. The lowest incidence occurred in December with an incidence of 0.16 infections per 100 admissions (Fig 2). World Health Organization’s core components of infection prevention and control were implemented in a step-wise manner after the detection of an outbreak. In 2014, a 1 year-long prevalence for CRKP in health-care settings in Barbados was 49.6 per 100,000 population and incidence of blood stream infection was 3.2 per 100,000 population.

## Discussion

Results of our study show that several factors were associated with an increased risk of acquisition of CRKP, including location in the High Dependency Unit, increased antimicrobial usage, and placement of urinary catheters. Elderly patients had a long length of stay within the hospital and often remained within the hospital for social care prior to transfer to limited capacity long care institutions, likely contributing to the prevalence of CRKP. To mitigate the challenge of the high prevalence of CRKP, the World Health Organization’s core components of Infection Control and Prevention and management of urinary catheters were implemented [18]. This study draws attention to a focus on management and use of urinary catheters in a resource-limited setting. Adherence to appropriate use can help to reduce catheter-associated urinary tract infections [19]. Training at all levels of healthcare workers was instituted to help define the indication and use of devices and urinary catheters for non-ICU patients. In Barbados, non-ICU patients often required urinary catheters due to benign prostate hypertrophy or after cerebrovascular accidents awaiting either surgical intervention or rehabilitation.

There is small but growing body of literature on carbapenemases from countries in the Caribbean including Cuba, Guadeloupe, Trinidad and Tobago, and Puerto Rico [20,21]. KPC-3 specifically has been documented in *Acinetobacter* species in Puerto Rico but not in other Caribbean nations [22]. This study adds to the minimal body of current literature on carbapenemases in the English-speaking Caribbean, and specifically is one of the first to address KPC and CRKP [21]. In addition, this study further highlights the presence and person-to person transmission of the dominant worldwide clone ST-258. Molecular epidemiology of the select viable samples showed a dominant clone and the possibility of person-to-person transmission within a healthcare setting.

Limitations of the 2013 point prevalence study include mortality data limited to the follow-up time within one month after the swab collection and tracking of antimicrobial use limited to the 14 days prior to swab collection. Resource limitations prevented the ability to analyze all positive isolates with PFGE. Lack of prospectively collected data prevented tracking of the colonization to infection ratio or other further metrics in the 2014 prevalence study.

As a result of the findings of this study a national action has been implemented for screening for CRKP with rectal swabs in all patients who are in the hospital for longer than a month. This study highlights, for one of the first times in literature, the spread of CRKP in long-term and acute care facilities patients in a resource limited setting. Patients who were colonized/infected were in the hospital longer, highlighting the impact of length of stay on risk of acquisition and person-to-person transmission. Resource limited settings like Barbados have a limited capacity of long-term care facilities (24 beds) leading to the intermingling of acute and long care based patients. This necessitates the screening of long care patients in these settings.

Surveillance cultures have been used to achieve successful reduction in CRKP infections [23,24]. Thus, admission and weekly rectal swab screening for patients in the HDU and all the ICUs in the hospital was initiated as these were recognized from the study to be high risk areas [10].

The use of antimicrobials (especially fluoroquinolones) was a predictor of rectal CRKP carriage based on this study. In the hospital, there were no policies guiding the use of carbapenems and there was no formal antimicrobial stewardship program (ASP). The hospital instituted an ASP which was highlighted in the January 2017 WHO AMR newsletter [25].

Tourism is one of the Caribbean's major economic sectors, with over 20 million visitors contributing $51.9 billion towards the area's gross domestic product in 2014, which represented 14.6% of its total GDP [26]. Barbados remains a top tourist destination and in 2014 saw 519,601 long stay visitors and 557,898 cruise ship passenger visitors [27]. The risk for transmission of AMR organisms across several continents to the Caribbean bears interest. The distribution of the major AMR organisms such as Methicillin-resistant *Staphylococcus aureus* (MRSA) clones in the French (Guadeloupe and Martinique) and non-French territories (Jamaica and Trinidad and Tobago) were shown to be different. The clones most closely resembled those found in the home countries of the travelers who visit the islands [28]. This implies the potential that the distribution of AMR is affected by tourist migration and further reveals the importance of this study.

Following initial findings, Barbados is establishing program consistent with the recently described approach of containment of resistant gram-negative organisms in low resource settings [29]. Measuring the prevalence of CRKP in Barbados has helped drive change in practice and policy at the hospital level through the start of a formal Infection Prevention and Control (IPC) program, at the national level via the commencement of a National IPC Committee, and in the Caribbean region through mentorship programs in IPC and ASP of other English-speaking Caribbean countries including Saint Vincent and the Grenadines, Saint Lucia, Grenada, and Saint Kitts and Nevis. Barbados, following the outbreak, started a regional IPC conference held yearly from 2014 with an aim to set up a regional surveillance system for AMR [25].
